# 

**Published:** 1880-06

**Authors:** 


### CONTENTS.

### COMMUNICATIONS.

On the Importance of the Teeth, in Reference to the Health.......... 249
Chemical Action on Tooth Bone and Fillings .......................... 256
Sun-Spots and Meteorology.......................................... 263
Clinic Reports.................................................... 266
Proceedings of the Twenty-First Annual Meeting of the Northern Ohio Dental Association.......................................... 269

### EDITORIAL.

A Directory of Dentists ............................................ 280
A Powerful Disinfectant............................................ 283
The Millers........................................................ 283
Of Individual Importance to every one Interested in Dentistry....... 284
Bibliographical.................................................... 286
Bibliographical.................................................... 288
Transactions....................................................... 288
Connecticut Valley Dental Society.................................. 289
Meeting ........................................................... 290
California State Dental Association ............................... 291
American Dental Association. ...................................... 291
Transactions....................................................... 291
Correction......................................................... 292
A New Medical Journal.............................................. 292
Obituary........................................................... 292

